# Case report: Metastatic choriocarcinoma in the second trimester of a viable pregnancy with successful delivery and outcome after chemotherapy

**DOI:** 10.3389/fonc.2024.1345011

**Published:** 2024-03-08

**Authors:** Yalin Tian, Jiayi Yu, Xin Dan, Tanglin Chen, Yalin He

**Affiliations:** ^1^ Department of Radiation Therapy and Chemotherapy for Cancer Nursing, West China Second University Hospital, Sichuan University, Chengdu, Sichuan, China; ^2^ Key Laboratory of Birth Defects and Related Diseases of Women and Children (Sichuan University), Ministry of Education, Chengdu, Sichuan, China

**Keywords:** gestational choriocarcinoma, metastases, viable pregnancy, chemotherapy, second trimester, case report

## Abstract

Metastatic choriocarcinoma during viable pregnancy is rare worldwide, and neonate survival following pregnancy termination in the second trimester is uncommon. Here, we report the successful delivery of a pregnancy by a patient with metastatic choriocarcinoma, who received three courses of etoposide, methotrexate, actinomycin D, cyclophosphamide, and vincristine (EMA-CO) chemotherapy in the second trimester. After multidisciplinary discussions, she was administered paclitaxel and carboplatin (TC) chemotherapy. Regular contractions occurred during her first paclitaxel infusion, and a healthy infant was delivered by cesarean section at 26^+4^ gestational weeks. Choriocarcinoma was not detected in the placenta. Following delivery of the pregnancy, the patient underwent total treatment comprising one cycle of TC, seven cycles of EMA-CO, and five courses of etoposide, cisplatin, methotrexate, and dactinomycin chemotherapy; her serum level of beta–human chorionic gonadotropin gradually fell after chemotherapy. Uterine and pulmonary metastases shrank, and no distant metastasis or recurrence were found until the eighth course of maintenance treatment with immunotherapy. The patient received periodic chemotherapy for recurrence at the time of publishing this case report. The child was disease-free 15^+^ months after delivery. Despite serious metastases and complications, metastatic choriocarcinoma diagnosed in the second trimester of pregnancy can be successfully treated with minimal delay by multidisciplinary medical and nursing management.

## 1 Introduction

Choriocarcinoma is a rare, highly aggressive, malignant trophoblastic neoplasm that is a histologic subtype of gestational trophoblastic neoplasia (GTN) affecting approximately 1 in 40,000 pregnancies in Europe and North America and 9.2 per 40,000 pregnancies in Southeast Asia (1). Coexistence of choriocarcinoma with an intrauterine pregnancy is associated with high mortality rates for both mother (62%) and fetus (65%) (2). In patients with choriocarcinoma, the fetus and accessory tissues are generally abnormal, and choriocarcinoma concurrent with a normal pregnancy is extremely rare, accounting for 1 in 160,000 pregnancies (3). Most patients with choriocarcinoma are diagnosed in the third trimester of pregnancy, and the pregnancy is immediately terminated (4–7). Because of their relatively low molecular weight, chemotherapy drugs can penetrate the placenta and exert adverse effects on fetuses, limiting their use during pregnancy; hence, exposure to chemotherapy during pregnancy is a great concern for mothers and physicians (8). Here, we report a patient with metastatic choriocarcinoma diagnosed in the second trimester of a viable pregnancy, who underwent chemotherapy and had a successful delivery. This case provides a clinical reference for treating and preserving pregnancy in patients with GTN.

## 2 Case presentation

The patient was a 27-year-old pregnant woman (gravida 2, para 0), who had previously undergone six courses of etoposide and cisplatin (EP) chemotherapy due to an “invasive mole” diagnosed at a local hospital in 2018. No vaginal bleeding or discharge were noted during early pregnancy. An ultrasound scan at 13^+6^ gestational weeks showed a cystic-solid mass between the lower edge of the placenta and the cervix. At 18^+1^ gestational weeks, color ultrasound examination indicated a metastasis of 6.48 cm × 6.68 cm × 7.63 cm on the right wall of the uterus, which was considered to be GTN. In June 2022, she was admitted at 18^+4^ gestational weeks, because of the uterine mass that had been present for more than 1 month. Pelvic magnetic resonance (MR) examination showed that the metastatic focus protruded under the serous membrane.

**Figure 1:**
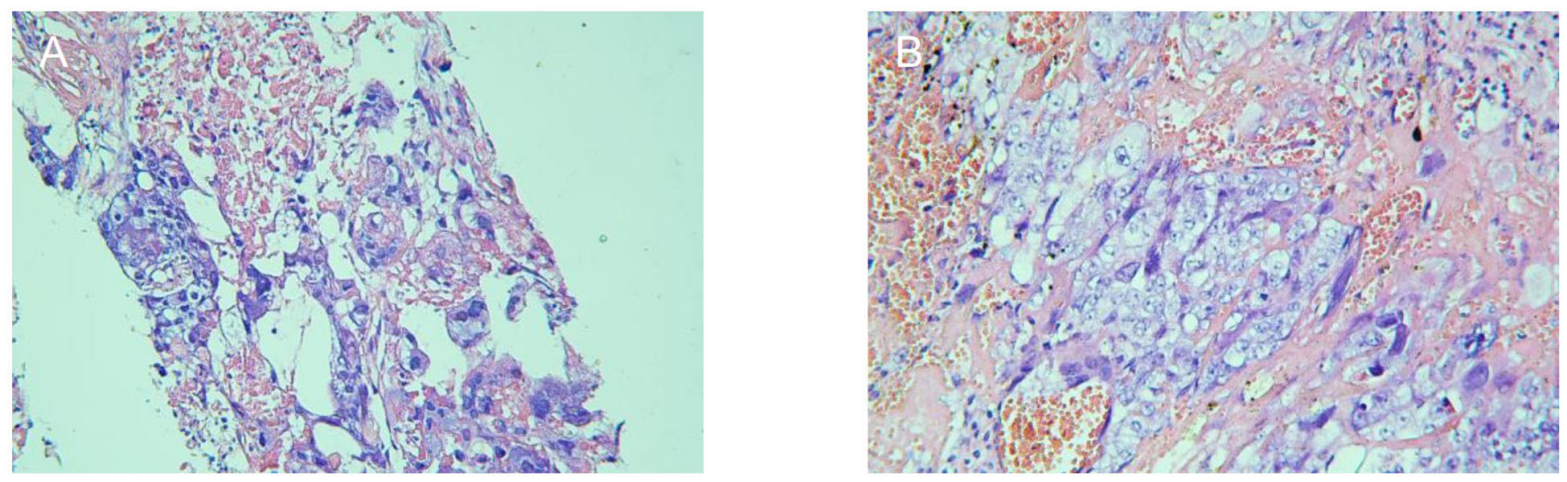
Pathological examination to confirm that the pulmonary metastasis **(A)** and uterine mass **(B)** were choriocarcinoma.

Five days after admission, chest MR examination indicated multiple pulmonary metastases (maximum size, 1.8 cm × 1.2 cm). Investigation by CT-guided needle biopsy of the right lung metastasis was conducted the following day. She experienced sudden hemoptysis, dyspnea, and thoracalgia during insertion of the CT-guided needle because of pneumothorax. After oxygen inhalation and supportive treatment, pneumothorax did not recur and the patient underwent successful biopsy 2 days later. Pathological examination of the pulmonary metastasis confirmed choriocarcinoma (**Figure 1A**). Continuing or terminating the pregnancy were both considered high-risk at 20 gestational weeks. The patient and her family decided to continue the pregnancy after being fully informed of the risks.

The tumor was FIGO stage III (prognostic score, 14); therefore, etoposide, methotrexate, actinomycin D, cyclophosphamide, and vincristine (EMA-CO) chemotherapy was given at 20^+3^ gestational weeks. After three cycles of chemotherapy, the chemotherapeutic effect was remarkable, resulting in shrinkage of the pulmonary metastases and a gradual decrease in beta–human chorionic gonadotropin (β-hCG) level; however, pelvic MR examination at 24^+3^ gestational weeks revealed that the myometrial metastasis was growing. As her condition was stable, the multidisciplinary team decided to switch the EMA-CO regimen to paclitaxel and carboplatin (TC) chemotherapy at 26^+3^ gestational weeks. Regular contractions occurred during the first paclitaxel infusion at 26^+4^ gestational weeks. Nifedipine was given to stop the contractions but failed, and the patient and her family refused any other tocolysis. Subsequently, an emergency cesarean section was performed under lumbar anesthesia. A live female infant weighing 640 g was delivered, with Apgar scores of 7, 8, and 8 at 1, 5, and 10 min after delivery. The placenta appeared normal and intact and pathological examination did not reveal any abnormality. The baby was transferred to the neonatal intensive care unit (NICU).

The patient was initially administered one cycle of TC chemotherapy 3 days after delivery and then EMA-CO chemotherapy twice a week. The size of the uterine mass shrank to 4.2 cm × 3.3 cm × 4.0 cm, and its boundary became clear after seven courses of postpartum chemotherapy. The gynecological surgeon considered the uterine mass resistant to chemotherapy and that hysterectomy was feasible. The patient underwent hysterectomy and histological assessment confirmed that the uterine mass was choriocarcinoma (**Figure 1B**). The EMA-CO regimen was switched to etoposide, cisplatin, methotrexate, and dactinomycin (EP-EMA) chemotherapy after one course of postoperative chemotherapy, because of an unsatisfactory decrease in β-hCG, and tislelizumab was added after one course of EP-EMA for the same reason.

**Figure 2:**
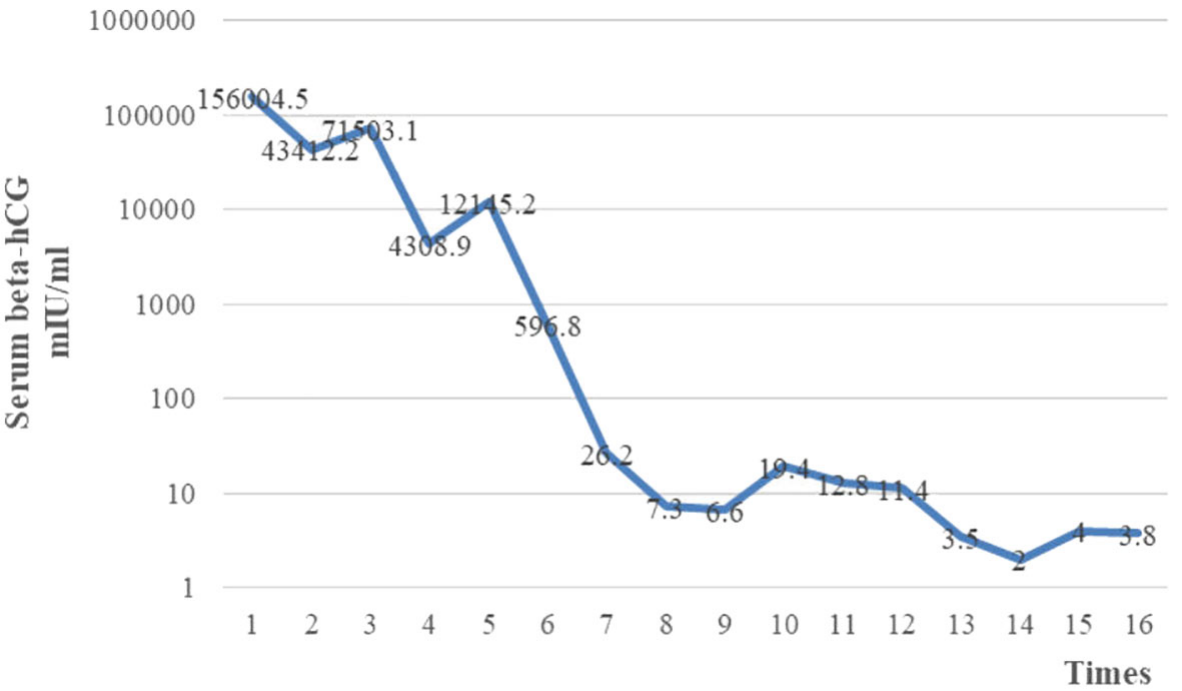
Serum β-hCG level returned to the normal range after eight cycles of chemotherapy.

**Figure 3:**
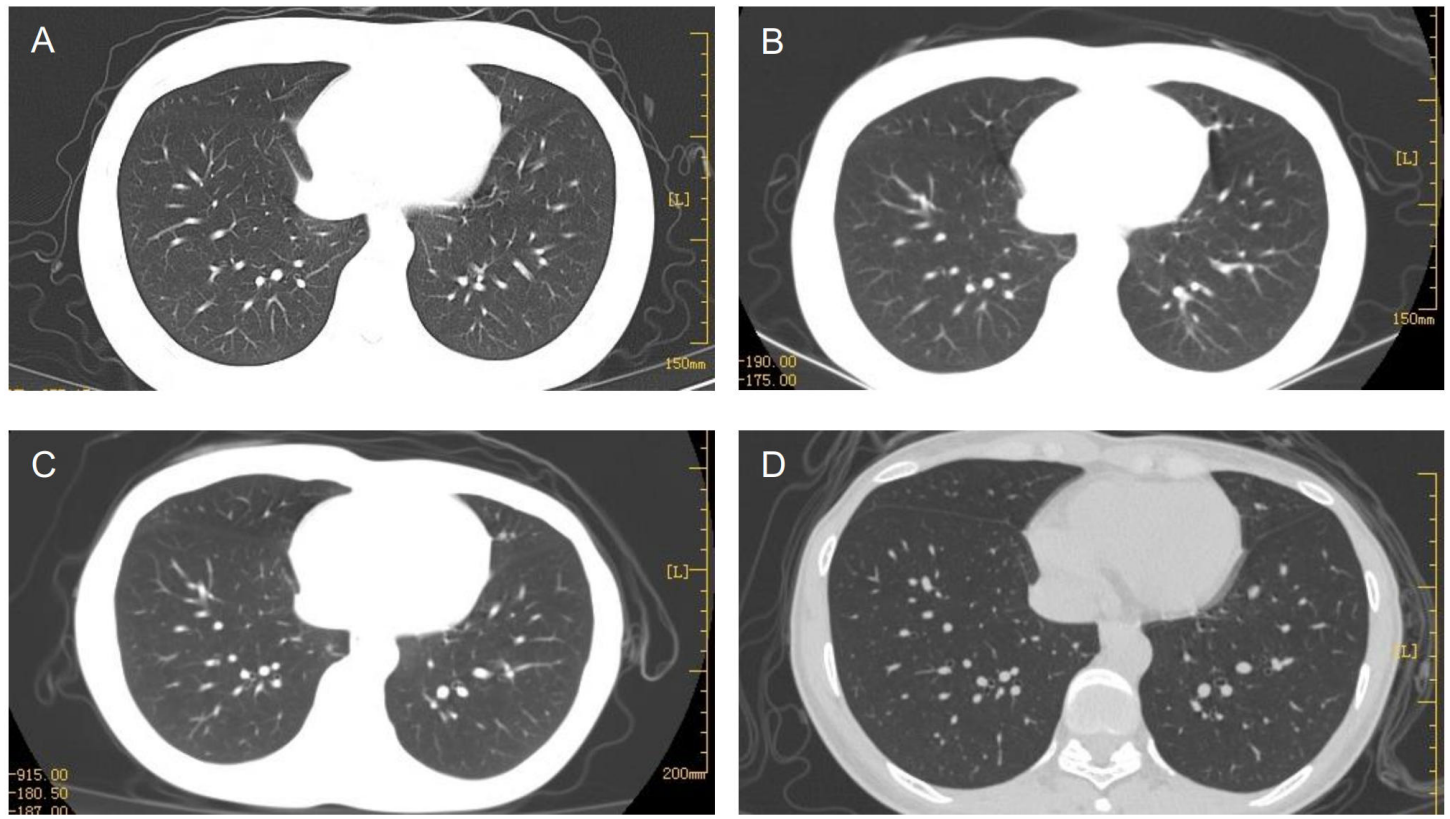
Multiple pulmonary metastases gradually subsided after six cycles **(A)**, 12 cycles **(B)**, and 3 months **(C)** of chemotherapy. The patient relapsed after eight maintenance treatments with tislelizumab **(D)**.

After four cycles of EP-EMA chemotherapy combined with tislelizumab, her β-hCG level returned to the normal range (**Figure 2**), and pulmonary metastases were reduced (**Figures 3A–C**). No distant metastasis or recurrence were found until the eighth course of maintenance treatment with tislelizumab (**Figure 3D**). The psychological condition of the patient was normal, based on assessment using the Hospital Anxiety and Depression Scale (HADS), which has excellent reliability and validity (9). The patient is undergoing periodic bleomycin, etoposide, and cisplatin (BEP) chemotherapy for recurrence. The baby was discharged 126 days after delivery weighing 2,530 g weight and was disease-free 15^+^ months after delivery.

The clinical course of the patient from initial diagnosis to last follow-up is illustrated in **Table 1**.

**Table 1:** Patient clinical course from initial diagnosis to last follow-up.

Time point	Events	Auxiliary examination	Treatment/Nursing
13^+6^ gestational weeks	Visit a local hospital	Ultrasound scan showed a cystic-solid mass between the lower edge of the placenta and the cervix.	Recommend to go to a higher-level hospital for treatment, because of limited local medical conditions
18^+1^ gestational weeks	Visit the outpatient department of our hospital	Color ultrasound indicated a metastasis of 6.48 cm × 6.68 cm × 7.63 cm on the right wall of the uterus, which was considered to be GTN.	Multidisciplinary discussions
18^+4^ gestational weeks	Admitted to hospital because of a uterine mass that had been present for more than 1 month	Serum β-hCG, 115,607–212,014 mIU/mL	Monitor fetal heart rate and fetal movement ECG monitoring Nutrition guidance
19^+3^ gestational weeks	Pneumothorax during CT-mediated needle biopsy of right lung metastasis	CT examination of the chest indicated pneumothorax Serum β-hCG, 126237.1 mIU/mL	Oxygen inhalation by mask Transfer to ICU
20^+3^ gestational weeks	Transfer to the Department of Radiation Therapy and Chemotherapy for Gynecological Cancer	Pathological examination of the pulmonary metastasis confirmed choriocarcinoma Serum β-hCG, 156004.5 mIU/mL	Psychological assessment and support Monitor fetal heart rate and fetal movement Peripherally inserted central catheter (PICC) EMA-CO chemotherapy twice a week
24^+3^ gestational weeks	Regular outpatient follow-up during chemotherapy	Pelvic MR examination revealed that the metastasis on the right wall of the uterus was growing, and the surrounding muscle layer was thinner than at the previous examination	Multidisciplinary discussions
26^+3^ gestational weeks	Condition stable	Serum β-hCG, 79,324.7 mIU/mL	Switch from EMA-CO to TC chemotherapy
26^+4^ gestational weeks	Regular contractions	Fetal monitoring revealed regular contractions	Immediately cessation of paclitaxel chemotherapy Nifedipine administration to prevent contractions Emergency cesarean section under lumbar anesthesia
3 days after delivery	Postpartum chemotherapy	Serum β-hCG, 4,308.9 mIU/mL	TC chemotherapy
28 days after delivery	Chemotherapy regimen changed	Serum β-hCG, 12,145.2 mIU/mL	EMA-CO chemotherapy twice a week
3^+^ months after delivery	Pelvic MR examination showed the uterine mass had shrunk to 4.2 cm × 3.3 cm × 4.0 cm, and the boundary was clear	Serum β-hCG, 6.6 mIU/mL	Hysterectomy EMA-CO chemotherapy twice a week
4^+^ months after delivery	Chemotherapy regimen changed	Serum β-hCG, 12.8 mIU/mL	EP-EMA chemotherapy twice a week
5^+^ months after delivery	Immunotherapy administered	Serum β-hCG, 11.4 mIU/mL	Tislelizumab administered twice a week EP-EMA chemotherapy twice a week
7^+^ months after delivery	Chemotherapy stopped and maintenance treatment administered	Serum β-hCG, 2 mIU/mL	Tislelizumab once every 3 weeks
12^+^ months after delivery	Disease recurrence	CT examination of the chest indicated new pulmonary metastases. Serum β-hCG, 26.1 mIU/mL	BEP chemotherapy once every 3 weeks

GTN, gestational trophoblastic neoplasia; β-hCG, beta–human chorionic gonadotropin; ECG, electrocardiogram; ICU, intensive care unit; EMA-CO, etoposide, methotrexate, actinomycin D, cyclophosphamide, and vincristine; MR, magnetic resonance; TC, paclitaxel and carboplatin; EP-EMA, etoposide, cisplatin, methotrexate, and dactinomycin; BEP, bleomycin, etoposide, and cisplatin.

## 3 Discussion

Choriocarcinomas can originate from either current or previous pregnancies (10), and it is difficult to determine their origin because most women with choriocarcinoma have a previous history of pregnancy. In some metastatic cases, choriocarcinoma has been detected in the placenta by histopathologic examination (11–14); however, it is not routine to conduct pathological assessment of the placenta (15). Short tandem repeat polymorphism (STR) analysis can provide more accurate determination of the origin of choriocarcinomas; for example, according to STR analysis, the choriocarcinoma in a patient described by Ding et al. may have originated from her most recent spontaneous abortion (14). We were unable to confirm the origin of the choriocarcinoma in our case as we did not conduct STR analysis.

Vaginal bleeding is the most common clinical manifestation of choriocarcinoma, but symptoms vary widely during pregnancy; for example, metastatic choriocarcinoma was found in a multiparous woman who initial presented with intractable lower back pain (16). The patient described in this study did not experience vaginal bleeding. Further, pulmonary metastases of choriocarcinoma were confirmed without delay after she was admitted. In previous reports, choriocarcinoma metastases have been found in the lung, uterus, brain, and vagina, and other tissues (10, 17), among which choriocarcinoma most often metastasizes to the lung, causing respiratory symptoms, such as cough, hemoptysis, and dyspnea, whereas choriocarcinoma with brain metastasis may cause neurological symptoms. Hence, choriocarcinoma should be considered when a pregnant woman presents with visceral metastases even without vaginal bleeding.

Chemotherapy is the main treatment for choriocarcinoma, with a cure rate of approximately 90%, even for metastatic disease (1); however, there is no consensus on appropriate treatment for choriocarcinoma concurrent with pregnancy, because of its rarity. It is considered better for patients in early pregnancy to terminate the pregnancy and receive standard chemotherapy, whereas antepartum chemotherapy has been suggested for those diagnosed in late pregnancy (10). Among cases described by Ding et al. (14), Bircher et al. (17), and Nabers et al. (18), who received antepartum chemotherapy during the second trimester, live fetuses were delivered after chemotherapy; however, no long-term follow-up of the newborns was conducted. The application of immunotherapy in refractory GTN has also become the subject of increasing attention, since Ghorani et al. (19) reported the first use of pembrolizumab in refractory GTN. The newborn reported in this study was disease-free 15^+^ months after delivery, even after exposure to chemotherapy in the second trimester. Tislelizumab was added during postpartum chemotherapy. Symptom management was conducted during chemotherapy and immunotherapy. The patient suffered from no side effects, other than mild nausea, vomiting, and myelosuppression. Therefore, we conclude that administration of chemotherapy during the second trimester is relatively safe and that symptom management plays a positive role during chemotherapy and immunotherapy for choriocarcinoma concurrent with pregnancy.

Most reports on choriocarcinoma concurrent with viable pregnancy documented that the patient terminated their pregnancy immediately after diagnosis, common reasons for which have included life-threatening symptoms of the mother and fetus and concerns about chemotherapy-induced fetal abnormalities (4–7, 11). Adjustment to the psychological transition from having a healthy pregnancy to a malignant choriocarcinoma is extremely challenging for patients and their families, who may experience negative emotions, such as sadness, anxiety, and shock, as well as isolation, fertility concerns, and worries about recurrence (12). A cross-sectional study in Australia indicated that depression and sexual dysfunction were the most important adverse effects of GTN (20). Our patient was encouraged to express her feelings, and, with the support of her family, no psychological issues were detected, based on her HADS scores each time she was admitted to our hospital.

## 4 Conclusion

Metastatic choriocarcinoma is extremely rare in women who are carrying a viable pregnancy, but our case emphasizes that it must be considered when a visceral metastasis is identified in a pregnant woman, even in the absence of vaginal bleeding. Pathological confirmation is essential for proper management of such cases. Chemotherapy, together with symptom management, is relatively safe during the second trimester and can improve survival of both the mother and fetus. Regular contractions and other related symptoms should be closely observed during chemotherapy, and the medical team should be ready for emergency cesarean section. Moreover, psychological support is important to help the patient and her family cope with the transition from healthy pregnancy to also having malignant choriocarcinoma.

## Data availability statement

The original contributions presented in the study are included in the article/supplementary material. Further inquiries can be directed to the corresponding author.

## Ethics statement

Written informed consent was obtained from the individual(s) for the publication of any potentially identifiable images or data included in this article.

## Author contributions

YT: Writing – original draft. JY: Writing – review & editing. XD: Writing – review & editing. TC: Writing – review & editing. YH: Writing – review & editing.
